# Irreparable Radial Nerve Palsy Due to Delayed Diagnostic Management of a Giant Lipoma at the Proximal Forearm Resulting in a Triple Tendon Transfer Procedure: Case report and Brief Review of Literature

**DOI:** 10.2174/1874325001711010794

**Published:** 2017-08-21

**Authors:** Ingo Schmidt

**Affiliations:** SRH Poliklinik Gera GmbH, Straße des Friedens 122, 07548 Gera, Germany

**Keywords:** Elbow joint, Radial nerve, Giant lipoma, Radial nerve palsy, Neurotmesis, Functional recovery, Triple tendon transfer

## Abstract

**Background:**

Non-traumatic radial nerve palsy (RNP) caused by local tumors is a rare and uncommon entity.

**Methods:**

A 62-year-old female presented with a left non-traumatic RNP, initially starting with weakness only. It was caused by a benign giant lipoma at the proximal forearm that was misdiagnosed over a period of 2 years. The slowly growth of the tumor led to an irreparable overstretching-related partial nerve disruption. For functional recovery of the patient, a triple tendon transfer procedure had to be performed.

**Results:**

Four months after surgery, the patient was completely able to perform her activities of daily living again. At the 10-months follow-up, strength of wrist extension, thumb's extension and abduction, and long fingers II-V extension had all improved to grade 4 in Medical Research Council scale (0-5). In order to restore motion, the patient reported that she would undergo the same triple tendon transfer procedure a second time where necessary. Due to the initially misdiagnosed tumor, there was an overall delayed duration of time for functional recovery of the patient.

**Conclusion:**

The triple tendon transfer procedure offers a useful and reliable method to restore functionality for patients sustaining irreparable RNP. However, it must be noted critically with our patient that this procedure probably would have been avoided. Initially, there was weakness only by entrapment of the radial nerve. RNP caused by local tumors are uncommon but known from the literature, and so it should be considered generally in differential diagnosis of non-traumatic RNP.

## 1 INTRODUCTION

Radial nerve palsy (RNP) is a severe injury mostly occurs by shaft fractures of the humerus and/or iatrogenic lesions by surgical procedures. In contrast, non-traumatic RNP is a rare entity, and it can be caused by various conditions. One of these conditions are local tumors potentially leading to nerve compression similar to a compartment syndrome. We present 1 case of a non-traumatic RNP caused by a giant lipoma (GL) at the proximal forearm that was initially misdiagnosed as mononeuropathy with unclear genesis by other treating physicians over a period of 2 years. The lesion led to a closed overstretching-related partial disruption (*i.e.* neurotmesis) of the radial nerve which required a triple tendon transfer procedure to restore the functionality of her wrist and hand. Tendon transfers for RNP were introduced in the late 19th century to restore functionality in patients who suffered from poliomyelitis, followed by further advances in World Wars I and II based on experiences of a large number of patients who sustained gunshot injuries at the upper extremity. Till today, over 50 variations in tendon transfers as a salvage option for the treatment of irreparable radial nerve lesions exist, and this procedure is an unchanged reliable and an excellent method [1]. A brief review of literature according to this procedure and also to the rare entity of GL will highlight this article.

## 2 CASE PRESENTATION

A 62-year-old female presented with a left RNP. At first presentation in our hospital, the patient reported progressive palsy starting with painless weakness only of her left wrist and long fingers extension 2 years ago. There was no history of any trauma or surgical procedure at her left upper extremity. Within these 2 years, the patient had a checkup by her family doctor and a neurologist. The diagnostic management included magnetic resonance imaging (MRI) of the cerebrum and cervical spine, electromyography, electroencephalography, and diagnostic analysis of blood and cerebrospinal fluid samples to exclude systemic specific or non-specific infectious or inflammatory or autoimmune diseases. No causes could be found that declared her RNP. So the diagnosis of a radial nerve mononeuropathy with unclear genesis was made, and the patient was treated by oral medication of glucocorticoids and vitamin B12. Additionally, the patient received a prefabricated wrist splint which immobilized only her left wrist in neutral position. Fortunately, stiffness or contractures in the wrist and all finger joints did not appear.

**Fig. (1):**
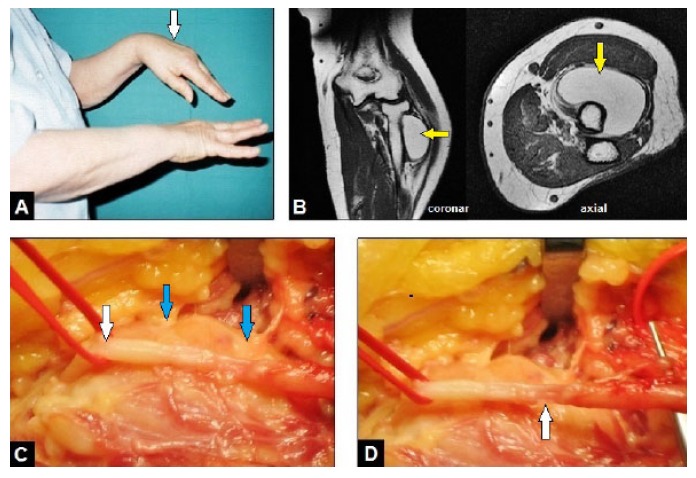
(Case presentation, initial and intraoperative findings according to the first procedure): **(A)** Clinical photograph of both forearms showing left (arrow) complete loss of wrist and thumb's extension and incomplete loss of extension in MCP joints II-V; **(B)** MRI demonstrating the monstrous GL (arrows) which surrounds the proximal radius shaft approximately a half part of its total circumference; **(C)** Clinical photograph showing the radial nerve (white arrow) which was fixed to the capsule of the GL (blue arrows); **(D)** Clinical photograph after monobloc removal of the entire GL and careful dissection of the radial nerve showing the overstretching-related partial disruption of the nerve with a gap of 1 cm involving a half part of its overall circumference (arrow).

On the first clinical examination in our hospital, an incomplete but severe RNP was present. The strength of extension of the wrist and thumb's extension and abduction was completely lost according to grade 0 in Medical Research Council (MRC) scale (0-5), and the extension in the metacarpophalangeal (MP) joints II-V showed palsy grade 3 in MRC scale (Fig. **1A**). The sensibility in the peripheral radial nerve area was completely lost. Electromyography revealed an advanced demyelinating radial nerve palsy, but not a complete neurotmesis. On physical examination, there was a painless and non-tender mass around the proximal radius. MRI revealed a submuscular, clearly demarcated and encapsulated tumor with a homogeneous and high-intensity signal similar to subcutaneous fat which surrounds the proximal radius shaft approximately a half part of its total circumference, and without visible septae inside the tumor after gadolinium injection (Fig. **1B**). According to these findings, the diagnosis of a benign GL was made by the radiologist, and surgical removal of the tumor with exploration of the radial nerve was detected by us. The tumor was exposed typically through a volar approach. The radial nerve was carefully dissected, it was fixed to the capsule of the GL (Fig. **1C**). The encapsulated GL with the size of 11x7x5 cm was completely removed in a monobloc manner. After that, an overstretching-related partial disruption of the nerve with a gap of 1 cm involving a half part of its overall circumference was visible (Fig. **1D**). At this time (*i.e.* 2 years after first neurological symptoms) a sural nerve graft would be inappropriate, and a tendon transfer procedure was detected by us. This could not go beyond the scope of this first surgical procedure because the time of tourniquet already lasted 80 minutes. Additionally, a complete neurotmesis of the radial nerve was not found by electromyography, and so the patient was not cleared up by us on specificities of a tendon transfer procedure preoperatively. Furthermore, the potential risk of problems with wound healing would have been too high due to the necessity of another large surgical incision at this time. The diagnosis of a benign GL was confirmed by histological examination.

**Fig. (2):**
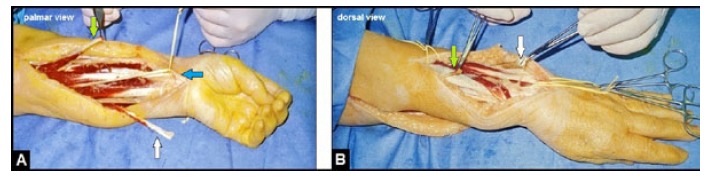
(Case presentation, intraoperative situs according to the Merle d'Aubigne and Lance procedure): **(A)** Clinical photograph showing dissection and distal detachment of the PT (green arrow), dissection and distal detachment of the FCU (white arrow), and dissection of the PL before its distal detachment (blue arrow), a large surgical incision was required for sufficient release of the longstanding intermuscular adhesions that is an essential prerequisite for its rerouting and well functioning gliding; **(B)** Clinical photograph showing the rerouted PL (green arrow) and FCU (white arrow).

Six weeks after removal of the GL, a triple tendon transfer procedure that included the transfer of the pronator teres (PT) to the extensor carpi radialis longus/brevis (ECRL/B), the transfer of the flexor carpi ulnaris (FCU) to the extensor digitorum communis (EDC), and the transfer of the rerouted extensor pollicis longus (EPL) to the palmaris longus (PL) was performed (Figs. **2A**, **B**). All tendon sutures were done in the Pulvertaft technique (end-to-side: PT to ECRL/B and FCU to EDC; end-to-end: EPL to PL). The wound healing was uneventful. The left upper extremity was immobilized in a plaster splint for 2 weeks. After that, the movement of elbow joint was freed, whereas the wrist was immobilized in the neutral position with a cast which additionally allowed the active flexion of all finger joints accompanied with its feather-related passive extension for another four weeks. Then, active strengthening of the wrist and all finger joints was started. Four months after surgery, the patient was completely able to perform her daily life activities again.

**Fig. (3):**
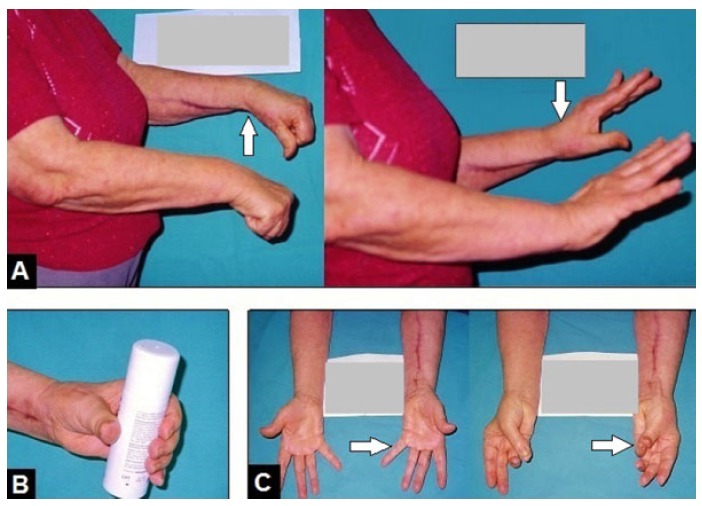
(Case presentation, 10-months follow-up): **(A)** Clinical photographs of both forearms showing left (arrow) good restoration of wrist and long fingers II-V extension; **(B)** Clinical photograph showing powerful object grasp; **(C)** Clinical photographs of both forearms showing left (arrows) complete restoration of thumb's extension and abduction, and sufficient thumb's circumduction with a powerful tip-to-tip pinch to the fifth finger.

At the 10-months follow-up, strength of wrist extension, thumb's extension and abduction, and long fingers II-V extension had all improved to grade 4 in MRC scale that was accompanied with a sufficient circumduction of the thumb (Figs. **3A**-**C**). The patient reported that she would undergo the same motion- restoring triple tendon transfer procedure again if it would be necessary.

## 3 DISCUSSION

Primary traumatic RNP is the most common injury associated with shaft fractures of the humerus with an incidence of 11,8% [2]. Secondary traumatic and/or iatrogentic radial nerve damage after surgery (open reduction and internal plate fixation, intramedullary nailing, total elbow arthroplasties) may occur in up to 32% of patients who undergo surgical treatments at the humerus [3, 4]. Iatrogenic RNP was observed in single cases after venipuncture, automatic blood pressure measurement, intramuscular injections and external fixation of the elbow as well [5-8]. Other causes for “trauma-related” RNP without an external injury or surgical intervention can be acute muscle compressions (*i.e.* “Sleep or Saturday night palsy”) similar to a compartment syndrome around the humerus, and spontaneous nerve torsion that is often associated with a segmental hourglass constriction of the radial nerve [9-13].

Isolated non-traumatic or “non-trauma-related” RNP is a very rare entity. It can be caused by cervical spine disorders (C7 radiculopathy), acute peripheral neuropathy (Guillain-Barré syndrome), neuromuscular disorders (distal myasthenia), acute viral infection (Herpes zoster), focal non-specific inflammations (myositis or synovitis of the elbow), specific inflammatory diseases such as rheumatoid arthritis or lupus erythematosus associated with Jaccuod's arthritis, amyloidosis, and systemic malignancy such as non-Hodgkin's lymphoma [14-24]. Another important cause for the pathogenesis of isolated non-traumatic RNP can be local radial nerve entrapment by benign or malign tumors arising from the bone, nerve, and soft tissue that was described in single cases only [25-28]. Allen *et al.* [29] reported a case series of 8 patients with GLs around the elbow joint (in one of them there was a well-differentiated liposarcoma in histological examination), and none of them had RNP or other nerve palsies. When a peripheral nerve entrapment is present, a surgical nerve decompression with neurolysis should be done as early as possible. It is also still widely known from the common peroneal nerve. The prognosis of functional recovery in patients with a common peroneal nerve palsy depends on severity of neurological deficit and the time of surgical neurolysis. In general, the prognosis for a demyelinating lesion is much more favorable than for an axonal loss lesion [30]. The outcome is usually favorable if surgery is done within 4 months after first presentation of neurological deficits, and less favorable in patients who have neurological symptoms for longer than 1 year [31, 32]. To our knowledge, our presented patient's case is the first report in the literature that describes RNP accompanied with an irreparable partial disruption of the nerve caused by a GL at the proximal forearm with an unacceptable delayed time in diagnostic management.

Lipomas arising from primordial adipocytes are the most common benign mesenchymal tumor of the upper extremity. However, lipomas with size greater than 5 cm in diameter are defined as GLs, and tumors of this size warrant a work-up for malignancy [33, 34]. They mostly occur in individuals between 50 and 60 years of age in the subcutaneous tissue of the head, neck, shoulders and back; but they are also observed in other locations [29, 35-37]. Multifocal appearance was described as well [38]. GLs in the hand usually grow slowly over the time of 1 year and longer, and can lead to soft tissue compression, nerve entrapment, tendon disruption, and limited joint motion when patients consult a physician too late and/or sufficient diagnostic management including early surgical revision has not been done by the treating physicians [38-43].

 They were found intermuscularly, intramuscularly, and interosseously [29]. The aetiology behind the appearance remains unclear, while their formation mechanism is often unknown to date; obesity, hypercholesteraemia, genetic triggers, and trauma associated with large and/or severe haematomas potentially leading to necrosis of fatty tissue followed by stimulation of pre-adipocytes are discussed [44-46]. When a GL is suspected, a liposarcoma must be ruled out. Liposarcomas are the most common soft tissue sarcomas, comprising up to 27% of all soft tissue sarcomas [47]. At the moment, MRI is the most imaging reliable method to differentiate lipomas from liposarcomas. Classically, lipomas appear as a homogeneous mass, with a sharp border, spontaneous T1 and T2 hypersignals, reduced signal intensity after erasure of the fat signal, and no raising in the signal using the gadolinium contrast agent [39], and an image that reveals heterogeneous mass, infiltration of the neighbouring tissues, and septae inside the tumor raises suspicion of a liposarcoma [44, 48].

 Histological subtypes of liposarcomas include well differentiated types accounting up to 45% of all types, myxoid types, round cell and pleomorphic types [29, 34]. Well-differentiated liposarcomas exhibit low malignant potential, myxoid liposarcomas display intermediate malignant behaviour, and round cell and pleomorphic liposarcomas exhibit aggressive behaviour with early metastasis [29]. GLs are treated by surgical removal. As the frequency of malignant transformation is extremely limited, the main reason for their removal is the disturbance they cause in the palm’s functionality and cosmetic appearance. Additionally, surgical resection of lipomas is the single treatment that allows liberation of the compressed nerve endings and effectively removes the tumor. Surgeons should perform a monobloc resection and a careful, safe dissection of the neurovascular branches to maximally reduce the risk of iatrogenic lesions. Regressions are extremely rare and are usually caused by the defective excision of the tumor. Surgical risks mainly concern postoperative infection, neural damage and the formation of a painful exuvia [39, 44]. Liposarcomas are notorious for local recurrence, and even with attempted total resection, local recurrence rates may be as high as 50% [29, 34].

**Fig. (4):**
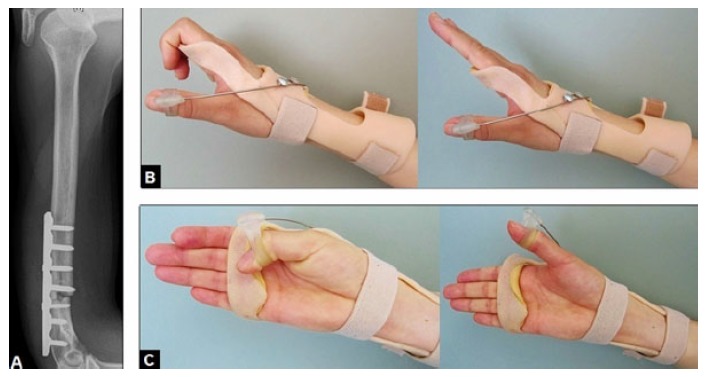
(Principle of our individually customized thermoplastic wrist and hand splint for patients sustaining RNP during both the duration of functional recovery with or without surgery and/or before tendon transfers): **(A)** Lateral radiograph of a 22-year-old female demonstrating open reduction and internal fixation of a right distal humerus shaft fracture performed in another hospital, there was an iatrogenic RNP postoperatively (axonotmesis); **(B)** Clinical photographs of the same patient showing that the wrist is immobilized in intrinsic plus position to avoid shortening of the antagonistic extrinsic flexor muscles in order to avoiding a secondary wrist flexion contracture, the MP II-V joints are immobilized in neutral position to avoid shortening of the antagonistic extrinsic flexor muscles of the long fingers in order to avoiding MP joint flexion contractures, the proximal interphalangeal joints are not immobilized because the intrinsic-related extension in these joints is not affected by RNP; **(C)** Clinical photographs of the same patient showing that the non-affected extrinsic-related flexion of the thumb is completely freed whereas the affected extrinsic-related extension and abduction of the thumb is passive realized by a flexible steel feather which is dorsally placed at the splint combined with alignment of the thumb in an abduction position, a spontaneous complete functional recovery was observed 7 months postoperatively.

When a partial or total disruption of the radial nerve is present such as in our case presentation, the mainly preferred options for surgical repair are autologous sural nerve grafting, tendon transfers, median to radial nerve transfers, and functional muscle transfers. The success of autologous sural nerve grafting depends on the duration of palsy and the age of patients. The problem of neurotmesis is that the extent of the Wallerian degeneration that takes place in these cases both in antegrade and retrograde directions cannot be estimated unequivocally. Recovery of radial nerve motor function may only be expected if this repair is carried out within 15 months after the injury [49]. Median to radial nerve transfers are indicated for older patients with medical comorbidities and for patients with significant hand stiffness which does not allow tendon transfers, and revealed good to excellent results when this procedure was done within a mean of 5,7 months after the injury [50]. Additionally, it is recommended to combine this procedure with simultaneous tendon transfer at the time of initial neurotization that offers earlier recovery of wrist extension and avoids some of the potential loss of the range of motion that can occur in some patients while nerve regeneration occurs [50]. The disadvantage is that this procedure always needs microsurgical expertise. Functional muscle transfers are detected when nerve or tendon transfers or neurotization becomes not possible [51]. For cases in which a flexion-contracture of the wrist is present, the total wrist fusion in neutral position is inevitable [52]. During the time of functional recovery after traumatic or non-traumatic RNP (*i.e.* before required or not required tendon transfers), our patients receive an individually customized thermoplastic wrist and hand splint to avoid flexion contractures in the wrist and finger joints (Figs. **4A**-**C**).

With our patient, the duration of RNP over 2 years did not allow more autologous sural nerve grafting. Fortunately, there were no contractures of the wrist and all other joints of the hand at first presentation in our hospital, and so that through tendon transfers, it became possible to restore hand function. A tendon transfer procedure is defined as relocation of the insertion of a functioning (*i.e.* antagonistic) muscle-tendon unit in order to restore lost movement and function at another site with irreparable nerve damage. The goals of this procedure are that it does not require microsurgical expertise, and the loss of function at the donor site is well compensated by the other synergistic muscle-tendon units. In 1898, Franke [53] was one of the first who described the transfer of the FCU to the EDC through the interosseous membrane to restore both wrist and long fingers extension in patients with RNP. In 1916, Jones [54] added the transfer of the PT to the ECRL/B for wrist extension, along with the transfer of the FCU to the long extensors of the ring and little finger, and the flexor carpi radialis (FCR) for index and thumb extension. In 1946, Zachary [55] recommended that the FCR should be preserved for wrist control. Over 50 variations in tendon transfers for surgical treatment of RNP have been described till today, which can be summarized mainly under 2 categories: First, the transfer of the PT to the ECRL/B for wrist extension combined with the FCU transfer for long fingers II-V extension and the PL transfer for thumb's extension; and secondly, the transfer of PT to the ECRL/B for wrist extension combined with the transfer of flexor digitorum superficialis (FDS) to EDC for long fingers extension that was first performed in 1960 by Boyes [56], and later modified by Chuinard *et al.* [57]. However, the Boyes technique is not free of any problems. The major problem with the transfer of the FDS is that the power of extension in MCP joints is not synergistic with finger flexor, which makes the motor retraining difficult following the transfer [58]. The loss of the FDS produces a relative overactivity of the extensor mechanism at the proximal interphalangeal joint potentially leading to a swan-neck deformity that requires surgical intervention when it impairs function after the Boyes procedure [59]. The triple transfer that was performed in our case is one of the most widely accepted combination originally described in 1946 by Merle d'Aubigne and Lance [60], and later modified by Riordan [61]. This modification includes the rerouting of the EPL to the PL and creates a viable combination both for extension and abduction of the thumb. The essential consideration is the avoidance of radial deviation of the wrist by centralizing the insertion of the ECRL [62]. The Merle d'Aubigne and Lance procedure is reported to be an excellent and reliable method; two retrospective studies revealed that 73,2 - 88,4% of treated patients were able to return in their original occupations 4 months after surgery [63, 64]. However, this procedure is also not free of any problems. When transferring the FCU, ulnar deviation with the wrist flexion is impaired. This is an important wrist movement, which is necessary for activities such as hammering and throwing [58]. Additionally, the ability to simultaneously extend the wrist and long fingers II-V cannot be achieved in most of patients treated by triple transfer procedures, whereas the majority of patients are able to move their fingers separately [63, 64].

## CONCLUSION

We present a patient's case with an irreparable RNP due to a benign GL at the left proximal forearm which was successfully treated by a triple tendon transfer procedure. However, it must be noted critically that this procedure probably would have been avoided. Initially, there was only weakness like a neurapraxie/axonotmesis due to entrapment of the radial nerve by the tumor. If at this time the GL would be diagnosed followed by removal of the tumor (*i.e.* decompression of the radial nerve), a spontaneous functional recovery of the radial nerve without any further surgical interventions would have been expected within 6 months to 1 year. Unfortunately, the aetiology of RNP was misdiagnosed by other treating physicians over a period of 2 years that led to the progression of RNP and resulted in an irreparable overstretching-related neurotmesis. In summary, RNP caused by local tumors is uncommon but known from the literature, and so it should be considered generally in differential diagnosis of RNP.

## LIST OF ABBREVIATIONS

ECRL/B: = Extensor Carpi Radialis Longus/Brevis
FCU: = Flexor Carpi Ulnaris
EDC: = Extensor Digitorum Communis
EPL: = Extensor Pollicis Longus
FCR: = Flexor Carpi Radialis
FDS: = Flexor Digitorum Superficialis
MRI: = Magnetic Resonance Imaging
MRC: = Medical Research Council
MP: = Metacarpophalangeal
PL: = Palmaris longus
PT: = Pronator Teres
RNP: = Radial Nerve Palsy
GL: = Giant Lipoma
